# multiSNV: a probabilistic approach for improving detection of somatic point mutations from multiple related tumour samples

**DOI:** 10.1093/nar/gkv135

**Published:** 2015-02-26

**Authors:** Malvina Josephidou, Andy G. Lynch, Simon Tavaré

**Affiliations:** Cancer Research UK Cambridge Institute, Li Ka Shing Centre, Robinson Way, Cambridge CB2 0RE, UK

## Abstract

Somatic variant analysis of a tumour sample and its matched normal has been widely used in cancer research to distinguish germline polymorphisms from somatic mutations. However, due to the extensive intratumour heterogeneity of cancer, sequencing data from a single tumour sample may greatly underestimate the overall mutational landscape. In recent studies, multiple spatially or temporally separated tumour samples from the same patient were sequenced to identify the regional distribution of somatic mutations and study intratumour heterogeneity. There are a number of tools to perform somatic variant calling from matched tumour-normal next-generation sequencing (NGS) data; however none of these allow joint analysis of multiple same-patient samples. We discuss the benefits and challenges of multisample somatic variant calling and present multiSNV, a software package for calling single nucleotide variants (SNVs) using NGS data from multiple same-patient samples. Instead of performing multiple pairwise analyses of a single tumour sample and a matched normal, multiSNV jointly considers all available samples under a Bayesian framework to increase sensitivity of calling shared SNVs. By leveraging information from all available samples, multiSNV is able to detect rare mutations with variant allele frequencies down to 3% from whole-exome sequencing experiments.

## INTRODUCTION

Somatic single nucleotide variants (SNVs) are point mutations found in the genomes of tumour cells, but not their matched normals. They often play important roles in tumour initiation, progression and metastasis by changing the amino acid sequence (missense mutation) or prematurely truncating encoded proteins (nonsense mutation). Discovering such cancer-related SNVs is confounded by the presence of millions of germline point mutations. To distinguish somatic from germline SNVs, it has become routine to sequence matched tumour-normal samples from the same individual.

Many cancer types are known to have a high degree of intratumour heterogeneity, so that the mutations present in a single tumour sample might not represent the full set of mutations in a particular cancer patient. As a result, multiregion sequencing studies, where multiple samples from the same individual are sequenced, are becoming increasingly popular.

Shah *et al*. (2009) assessed the mutational spectrum in a patient with metastatic lobular breast cancer and identified that out of 32 coding mutations, 19 were specific to metastases (1). Similarly, Yachida *et al*. (2010) compared somatic mutations in seven patients with metastatic pancreatic cancer and inferred that a mean of 36% of mutations were ‘progressor’ mutations not detectable across all samples of the same patient (2). Gerlinger *et al*. (2012) performed whole-exome sequencing on multiple samples from patients with metastatic clear cell renal carcinoma and inferred a branched model of tumour evolution with approximately 31% to 37% of somatic mutations common to all tumour samples (3). Similarly, Zhang *et al*. (2014) performed whole-exome sequencing on 48 tumour regions from 11 localized lung adenocarcinomas and found that 76% of all mutations were common to all regions of the same patient (4). De Bruin *et al*. (2014) sequenced 25 regions from seven nonsmall cell lung cancer patients and assessed how the poor prognosis of this cancer type might be linked to intratumour heterogeneity and divergent genomic instability processes (5).

Analysis of multiregion sequencing poses a new challenge for somatic variant calling, as both somatic sites and the distribution of somatic mutations across related samples need to be identified. Accurate and sensitive detection of the regional distribution of mutations can be key to reconstructing the evolution of a tumour, so somatic variant callers need to have enough power to identify rare mutations, without overcalling events.

### Calling SNVs from related samples

Somatic SNV calling has rapidly evolved over the past 5 years or so, from simple approaches based on hard thresholding on the number of variant reads, to fully probabilistic models. These probabilistic approaches report confidence scores by directly modelling the uncertainty in the sequencing data. This uncertainty is the result of Polymerase chain reaction (PCR) errors, contamination from normal cells, sequencing artefacts and mapping errors as well as the limited depth of coverage. The premise of sophisticated somatic variant callers is to allow optimal prediction of somatic variation at low depth of coverage and low variant allele frequencies.

Some of the first probabilistic SNV callers such as (6) called mutations in tumour and matched normal samples separately and identified somatic SNVs *post-hoc* by excluding mutations found in both the tumour and its matched normal. These were succeeded by their Bayesian equivalents, which jointly inferred mutations in tumour-normal pairs via a probabilistic model that accounted for the correlation between two samples from the same patient (7).

Even though there are now several excellent pairwise somatic variant callers, none of them can jointly analyse multiple related tumour samples. These are therefore analysed either independently using one tumour-normal pair at a time (5), or by using GATK's UnifiedGenotyper (8). The former method is inconvenient as it results in making multiple redundant analyses of the normal sample and requires merging different output files. The latter approach is not ideal as GATK's UnifiedGenotyper is specifically tuned to detect germline rather than somatic mutations from multiple unrelated samples (9,10). Most importantly, none of the approaches can easily be adapted to account for known patterns of relatedness between samples from the same patient.

We argue that fully Bayesian approaches can improve somatic variant calling from multiple related samples, in a similar manner that these approaches have improved somatic variant calling from matched tumour-normal pairs compared to independent sample analysis. In this contribution, we discuss the advantages and challenges of extending the current Bayesian paradigm to multiple same-patient samples. As a proof of principle, we present multiSNV, a first version of a somatic variant caller that extends pairwise analysis of tumour-normal pairs to joint analysis of multiple samples from the same patient.

multiSNV calls somatic SNVs across all available same-patient samples without pooling reads. It is based on a Bayesian framework that captures the relatedness between samples by modelling the probability of a mutation in a given sample, conditioned on the somatic status of all other samples. We present the statistical model, benchmark it on simulated and real data and compare performance to alternative workflows. We find that when multiple tumour samples are available, multisample variant calling can identify validated mutations with higher sensitivity.

## MATERIALS AND METHODS

### Modelling a multisample variant caller

Most current (pairwise) somatic variant callers are based on some approximation of the joint distribution of the tumour and normal genotypes, *P*(*G*^*N*^, *G*^*T*^), derived by directly modelling the dependency between the tumour and normal sample, *P*(*G*^*T*^|*G*^*N*^). A Bayesian approach to multisample variant calling should similarly try to infer the joint distribution of genotypes over *n* tumour samples and the normal, $P(G^{N},G^{T_{1}}, \dots , G^{T_{n}})$. Since tumour samples often consist of cells with different genotypes and unknown copy number, we argue that modelling the joint distribution of allelic compositions is more appropriate. The allelic composition of a patient sample denotes the aggregate set of alleles detected at a genomic locus, so when the allelic composition in a tumour sample, $S^{T_{i}},$ does not match the allelic composition in the normal, *S*^*N*^, the site may be treated as an SNV candidate.

### Statistical model used in multiSNV

In this first version of a multisample somatic variant caller, we infer the most likely set of allelic compositions $\lbrace S^{N},S^{T_{1}}, \dots , S^{T_{n}}\rbrace$ by using Gibbs sampling to approximate the posterior distribution $P(S^{N},S^{T_{1}}, \dots , S^{T_{n}}|\mathcal {D})$. $\mathcal {D}$ denotes the sequencing data (read bases with their corresponding base qualities) from the normal sample and *n* tumour samples so that at a particular chromosomal locus, $\mathcal {D}=\lbrace \mathcal {D}^{N},\mathcal {D}^{T_{1}}, \dots , \mathcal {D}^{T_{n}}\rbrace$.

To implement Gibbs sampling, we initialize the allelic compositions to random values and then draw a value for the allelic composition of each sample from its corresponding conditional distribution. Each newly drawn allelic composition value is used to update the conditional distributions of the rest of the samples and this procedure is repeated for a large number of iterations to allow the Markov chain to converge. Upon convergence, samples drawn from the conditionals may be assumed to come from the required joint distribution. We then infer as a point estimate of each allelic composition *S*, the most frequently drawn state.

A list of parameters used to describe the inference model in multiSNV is given in Table 1.

**Table 1.** List of parameters used to describe the inference model in multiSNV

Parameter	Description	Comments
*n*	Number of tumour samples	User-specified
*S*^*N*^	Allelic composition in normal sample	Estimated (one to two alleles allowed)
$S^{T_{i}}$	Allelic composition in tumour sample *i*	Estimated (one to three alleles allowed)
*k*	Denotes sample *k*	*k* ∈ {*N*, *T*_1_…*T*_*n*_}
*M*^*k*^	Number of alleles in allelic composition *S*^*k*^	Computed from inferred *S*^*k*^
$\mathcal {D}$	Pileup of bases and corresponding base qualities in all samples	Read from pileup file
$\mathcal {D^{N}}$	Pileup of bases and corresponding base qualities in normal sample	Read from pileup file
$\mathcal {D}^{T_{i}}$	Pileup of bases and corresponding base qualities in tumour sample *i*	Read from pileup file
$r^{k}_{j}$	Allele supported by read *j* in sample *k*	Read from pileup file
$e^{k}_{j}$	Error probability of read *j* in sample *k*	Read from pileup file
$v^{k}_{\tau }$	Total number of reads in sample *k* that support allele τ	Read from pileup file
${\boldsymbol f}^{\,k}$	The probability distribution of alleles in sample *k*	Estimated by maximizing $\mathcal {F}(g|D^{k},S^{k},{\boldsymbol a}^{k})$
${\boldsymbol a}^{k}$	Vector of shape parameters of Dirichlet prior on ${\boldsymbol f}^{\,k}$	Uniform prior
μ	Mutation rate	User-specified (default is 3 × 10^−7^)
$\mathcal {N}$	The set of values of *S*^*N*^ with nonzero prior probability	All monoallelic and diallelic compositions (total of 10)
ϕ_*z*_	Sampling probability of allelic composition $z$	Integrated out
δ_*z*_	Parameters of Dirichlet prior on ϕ_*z*_ of $S^{T_{i}}$	As described in Materials and Methods
$\delta _{z}^{N}$	Parameters of Dirichlet prior on ϕ_*z*_ of *S*^*N*^	As described in Materials and Methods
${c^{T_{i}}_{z}}$	Number of tumour samples excluding *T*_*i*_ with allelic composition $z$	From most recent draw of the Gibbs sampler
*n*_*z*_	Number of tumour samples with allelic composition $z$	From most recent draw of the Gibbs sampler
$w$	Scales pseudocounts for Dirichlet prior of allelic compositions	10 × (*n* + 1)

multiSNV analyses each location in the genome independently, so these parameters refer to a single genomic locus.

#### Modelling the conditional distributions

The conditional probability distribution of the allelic composition in the normal sample *S*^*N*^ is given by the product of the likelihood of the sequenced reads at a locus and the prior probability of observing *S*^*N*^ given the allelic composition in the tumour samples:

\begin{equation*} P(S^{N}|S^{T_{1}}, \dots , S^{T_{n}},\mathcal {D}) \propto P(\mathcal {D}|S^{N},S^{T_{1}}, \dots , S^{T_{n}}) P(S^{N}|S^{T_{1}}, \dots , S^{T_{n}}) \nonumber \end{equation*}

We assume conditional independence of the form:

\begin{eqnarray*} P(\mathcal {D}|S^{N},S^{T_{1}}, \dots , S^{T_{n}}) = P({\mathcal {D}^{N},\mathcal {D}^{T_{1}}, \dots , \mathcal {D}^{T_{n}}|S^{N},S^{T_{1}}, \dots , S^{T_{n}}}) \nonumber \\ = P(\mathcal {D^{N}}|S^{N},S^{T_{1}}, \dots , S^{T_{n}}) \prod \limits _{i=1}^{n} P(\mathcal {D}^{T_{i}}|S^{N},S^{T_{1}}, \dots , S^{T_{n}}) \nonumber \end{eqnarray*}

We also assume that the likelihood of the sequencing data in patient sample *k* depends only on the inferred sample allelic composition, so that:

\begin{equation*} P(D^{k}|S^{N}, S^{T_{1}}, \dots , S^{T_{n}}) = P(D^{k}|S^{k}) \end{equation*}

It follows that the conditional probability distribution of the allelic composition in the normal sample is given by:

\begin{equation*} P(S^{N}|S^{T_{1}}, \dots , S^{T_{n}},\mathcal {D}) \propto P(D^{N}|S^{N})P(S^{N}|S^{T_{1}}, \dots , S^{T_{n}}) \nonumber \end{equation*}

Similarly, the conditional probability distribution of the allelic composition in tumour sample *i* is given by:

\begin{equation*} P(S^{T_{i}}|S^{-T_{i}},S^{N},\mathcal {D}) \propto P(\mathcal {D}^{T_{i}}|S^{T_{i}})P(S^{T_{i}}|S^{N},S^{-T_{i}}) \nonumber \end{equation*}

where $S^{-T_{i}}$ denotes the set of all tumour allelic compositions except $S^{T_{i}}$. The following subsections describe our choice of likelihood and priors.

#### Likelihood model

The pileup of aligned reads that cover a particular chromosomal location in patient sample *k* is represented by vector ${\boldsymbol r}^{k}$, where each element $r^{k}_{j}$ of the vector is ∈ {*A*, *C*, *G*, *T*}. Each base has an associated base quality that gives the overall probability that the base has been miscalled. In our model, we assume that all three base calling errors are equally probable, so that if for example, $r^{k}_{j}=A$, the overall probability that the base has been miscalled as *A* is $e^{k}_{j}$, and the probability that the base has been miscalled as *A* given that it is *C*, *G* or *T*, is uniform. It is convenient to define

\begin{eqnarray*} \mathcal {G}^{k}= \biggr \lbrace (g_{A},g_{C},g_{G},g_{T}): g_{i} \ge 0, g_{A}+g_{C}+g_{G}+g_{T}=1, \nonumber \\ g_{i}=0 \hspace{5.69046pt} \text{if} \hspace{2.84544pt} i \notin S^{k} \biggr \rbrace \nonumber \end{eqnarray*}

The probability distribution of alleles in sample *k* is denoted by ${\boldsymbol f}^{\,k} \in \mathcal {G}^{k}$. The probability of base $r_{j}^{k}$ being called as τ ∈ {*A*, *C*, *G*, *T*} is

\begin{equation*} P(r^{k}_{j}=\tau |S^{k},{\boldsymbol f}^{\,k})= (1-e^{k}_{j})f^{k}_{\tau }+\frac{e^{k}_{j}}{3}(1-f^{k}_{\tau }) \end{equation*}

Since the bases $r^{k}_{j}$ are independent, the likelihood of the sequencing data *D*^*k*^ is given by the product of the individual likelihoods:

\begin{equation*} P(\mathcal {D}^{k}|S^{k},{\boldsymbol f}^{\,k})= \prod \limits _{{j}} P(r^{k}_{j}|S^{k},{\boldsymbol f}^{\,k}) \end{equation*}

#### Estimating ${\boldsymbol f}^{\,k}$

To estimate the probabilities ${\boldsymbol f}^{\,k}$ we compute the maximum-a-posteriori estimate from the posterior density $\mathcal {F}(g|D^{k},S^{k},{\boldsymbol a}^{k}),$ $g \in \mathcal {G}^{k}$, assuming a flat Dirichlet prior on the nonzero elements of $\mathcal {G}^{k}$, parameterized by ${\boldsymbol a}^{k}.$

For the normal sample, nonzero $a^{k}_{\tau }$ are set to about five times the median normal coverage as we want to bias our ratio towards allele fractions of 1 for homozygous sites and 0.5 for heterozygous sites. For tumour samples, we use a weaker prior with all nonzero $a^{k}_{\tau }$ set to around 20% of the median coverage in tumour samples.

We compute the likelihood $P(\mathcal {D}^{k}|S^{k},g)$ in the limit $e^{k}_{j} \rightarrow 0$, expecting this to be a good approximation as low quality bases are filtered out. Under these assumptions, the posterior $\mathcal {F}(g|D^{k},S^{k},{\boldsymbol a}^{k})$ is Dirichlet, parameterized by $a^{k}_{\tau }+v^{k}_{\tau }$ where $v^{k}_{\tau }$ is the total number of reads in sample *k* that support allele τ. This gives:

\begin{eqnarray*} {f^{k}_{\tau }=} \left\lbrace \begin{array}{ll}\displaystyle\frac{v^{k}_{\tau }+a^{k}_{\tau }-1}{\sum \limits _{ \tau \in S^{k}} (v^{k}_{\tau } + a^{k}_{\tau })-M^{k}}, & \tau \in S^{k} \\ 0, & \tau \notin S^{k} \end{array}\right. \end{eqnarray*}

where *M*^*k*^ is the number of alleles in the allelic composition *S*^*k*^.

The explicit modelling of the probability distribution of alleles τ ∈ {*A*, *C*, *G*, *T*} allows us to represent tumour samples as a heterogeneous mixture of cells with potential copy number aberrations. This improves sensitivity of detecting rare variants or variants at sites with altered ploidy.

#### Modelling the prior probability of each tumour allelic composition

We approximate the distribution of tumour allelic compositions $\lbrace S^{T_{1}}, \dots , S^{T_{n}} \rbrace$ as a multinomial sample from a universe with *k* = 2^4^ − 2 types (all allelic compositions with one to three alleles), and sampling probabilities ϕ_1_, …, ϕ_*k*_. The ϕ_*z*_ have a Dirichlet prior parameterized by δ_*z*_, defined according to how close a tumour allelic composition $z$ is to the inferred normal allelic composition *S*^*N*^. We set the parameter δ_*z*_ to $w$μ, whenever the allelic composition $z$ is one mutation away from *S*^*N*^, or in other words when $z$ has one extra nucleotide compared to *S*^*N*^. Since this corresponds to a somatic SNV at the locus, the default value of μ is set to 3 × 10^−7^, which is close to the estimated somatic mutation rate. The parameter $w$ is a weighting factor. For all other allelic compositions (excluding $z$ = *S*^*N*^), we set δ_*z*_ to $w$μ^2^, as we consider these transitions to be less likely. We set δ_*z*_ for $z$ = *S*^*N*^ so that $\sum \limits _{ z} \delta _{z}= w$. The prior probability of each tumour allelic composition is:

\begin{equation*} P(S^{T_{i}}=z|S^{-T_{i}},S^{N})= \frac{{c^{T_{i}}_{z}}+ \delta _{z}}{\sum \limits _{ z}{(c^{T_{i}}_{z}}+\delta _{z})} \nonumber \end{equation*}

where $c^{T_{i}}_{z}$ is the number of times allelic composition $z$ has been observed in the set of tumour allelic compositions excluding $S^{T_{i}}$. As $c^{T_{i}}_{z}$ increases, $P(S^{T_{i}}=z|S^{-T_{i}},S^{N})$ also increases, so that the algorithm has more power to detect shared mutations. To avoid overcalling these shared events, the weighting factor, $w$, is set to 10 × (*n* + 1), where *n* is the number of tumour samples.

#### Modelling the prior probability of the normal allelic composition

We use a similar approach to model the prior probability of *S*^*N*^, assuming the normal sample is pure and diploid. We denote the set of allelic compositions with one or two alleles by $\mathcal {N}$ and let:

\begin{equation*} {P(S^{N}=z|S^{T_{1}}, \dots , S^{T_{n}})=} \left\lbrace \begin{array}{ll}0, & z \notin \mathcal {N} \\ \frac{{n_{z}}+ \delta _{z}^{N}}{\sum \limits _{ z \in \mathcal {N} }{(n_{z}}+ \delta _{z}^{N})}, & z \in \mathcal {N} \end{array}\right. \end{equation*}

where *n*_*z*_ is the number of tumour samples with allelic composition equal to $z$, and $\delta _{z}^{N}$ is the parameter of the Dirichlet prior on the sampling probability ϕ_*z*_, where $z \in \mathcal {N}$. These parameters are defined according to how close an allelic composition $z$ is to the reference allele:

\begin{eqnarray*} {\delta ^{N}_{z}=} \left\lbrace \begin{array}{ll}w\alpha , & \hbox{heterozygous reference} \\ w\beta , & \hbox{heterozygous variant} \\ w\gamma , & \hbox{homozygous variant} \\ w\delta , & \hbox{homozygous reference} \end{array}\right. \end{eqnarray*}

with values defined as suggested in (11), following (12), so that *α* = 3.34 × 10^−4^, *β* = 8.33 × 10^−8^, *γ* = 1.665 × 10^−4^, *δ* = 0.9985. The weighting factor $w$ is similarly set to 10 × (*n* + 1).

### Software package

The multiSNV software package has been implemented in C++ and is available at http://www.compbio.group.cam.ac.uk/software/multisnv

## RESULTS

### Benchmarking multiSNV

We evaluated multiSNV using both simulated datasets and real exome-sequencing data from a validated multiregion sequencing study on clear-cell renal carcinoma (3). We benchmarked the performance of multiSNV against four other variant callers: SomaticSniper, MuTect, UnifiedGenotyper and Platypus as these represent well the currently available options for analysing multiple related samples. SomaticSniper (13) and MuTect (14) call somatic SNVs from tumour-normal pairs, so analysis of multiple tumour samples requires calling variants on each tumour-normal pair independently and then combining calls. In contrast, both Platypus (15) and UnifiedGenotyper (9,10) can call variants from multiple samples, but neither has been designed to address the challenges of calling somatic variants from matched tumour/normal samples.

### Simulation study

We simulated reads from four matched tumour samples and a normal, under a model of uniform sequencing depth with perfectly aligned reads and independent sequencing error, using SimulateReadsForVariants from GATK. The probability of sampling a variant allele was set to the variant allele frequency, and sequencing noise was added at the base quality rate. We ran multiSNV and the four other variant callers using settings close to default––details may be found in the Supplementary Material.

#### Improved sensitivity of calling shared somatic events

**Figure 1.**
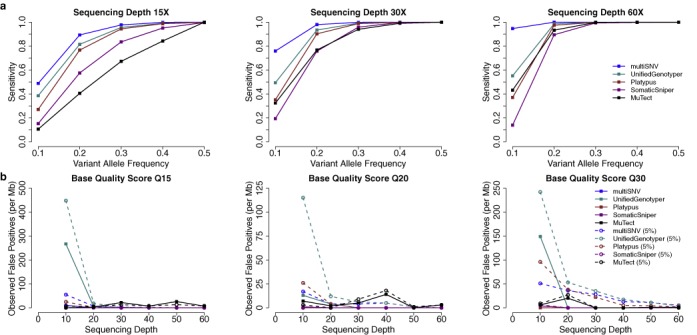
(**a**) Sensitivity in detecting shared mutations of increasing variant allele frequency at three different sequencing depths, using simulated reads of base quality score Q30. (**b**) Observed false-positive rate per megabase as a function of sequencing depth in the presence of 0 and 5% tumour contamination in the normal. Reads were simulated under a model of independent and uniform sequencing error, introduced at base quality rates of Q15, Q20 and Q30.

We simulated reads for the simple case where SNVs are found in all tumour samples at the same frequency. We computed the sensitivity of each variant caller, given by the ratio of true positive calls to the total number of simulated SNVs (3000). Reads were simulated with base quality score of Q30 and the simulation experiment was repeated for variant allele frequencies between 0.1 and 0.5 and sequencing depths between 15 × and 60 ×. We ran multiSNV and compared its sensitivity to the two other multisample callers (Platypus and UnifiedGenotyper) and the two pairwise somatic variant callers (SomaticSniper and MuTect). As shown in Figure 1, the three multisample callers outperform SomaticSniper and MuTect, and multiSNV is consistently more sensitive than all other methods. This is particularly evident at low allele frequencies. For example, at a variant allele frequency of 0.1 and sequencing depth 60, multiSNV outperforms the second most sensitive variant caller by more than 70%. This suggests that even though joint analysis of multiple samples enhances sensitivity compared to independent pairwise analyses, there are clear advantages to a multisample caller that is tailored to detect somatic variation from related tumour samples.

#### Robustness to independent sequencing noise

To confirm that the enhanced sensitivity of multiSNV is not at the expense of a high false-positive rate, we assessed the robustness of multiSNV to independent sequencing noise at default settings. This was done by simulating reads with Phred-scaled base qualities of 15, 20 and 30, and setting the variant allele frequency to 0, for different sequencing depths. We computed the false-positive rate, defined as the ratio of the number of false-positive calls to the total number of simulations (100 000).

False-positive rate results for all five variant callers for sequencing depths between 10 and 60 are shown in Figure 1. Most variant callers handle this type of sequencing error well, but multiSNV is one of the better performing with a false-positive rate lower than 10 per megabase, even at very low base qualities (Q15) and low sequencing depth (10). It drops towards 0 as noise levels decrease or sequencing depth increases.

#### Robustness to contamination of the normal by tumour cells

We also tested robustness to false positives when the normal sample is contaminated by tumour cells, by setting the variant allele frequency in all tumour samples to 0.5 and introducing 5% contamination in the normal sample. The results of this simulation experiment are summarized in Figure 1. multiSNV has some inherent robustness to contamination of the normal (we bias the normal to be homozygous reference and the expected allele fractions in the normal to be close to 0.5 or 1); however, the likelihood model itself does not explicitly account for any possible contamination from tumour cells. This implies that all variant reads in the normal are attributed to random sequencing error. It follows that the false-positive rate is higher when simulated reads have higher base qualities, as the probability of observing these high-quality variant reads is very low if the normal is homozygous reference, leading to more false-positive calls. We note that if there is significant contamination in the normal, the likelihood model should be extended accordingly.

### Clear-cell renal carcinoma data

Simulations fail to capture the noise and complexity of real datasets, so we also evaluated performance of multiSNV using the two datasets from (3) where exomes from multiple tumour samples of two patients with metastatic clear cell carcinoma were sequenced. In patient 1, we had access to whole-exome sequencing data from 11 spatially separated samples, of which three came from distant metastases, seven came from the primary tumour collected after nephrectomy and one came from germline DNA. In patient 2, we used whole-exome sequencing data from eight tumour samples of which two came from metastases, five came from the primary tumour and one came from germline DNA.

Originally, SNVMix2 had been used to call somatic variants and the regional distribution of a subset of nonsynonymous, coding SNVs was verified using Sanger sequencing. We ran multiSNV, SomaticSniper, MuTect, UnifiedGenotyper and Platypus and compared their overall number of calls, concordance with dbSNP and sensitivity in correctly detecting the regional distribution of the validated SNVs. We did not compare performance to SNVMix2 as this method analyses samples independently instead of as tumour-normal pairs.

#### Somatic variant analysis

We ran all tools using the settings described in the Supplementary Material (default unless otherwise stated). Only sites with nonzero depth in all samples were considered in the analysis. Approximately 2.7% of somatic events called by multiSNV involved a germline heterozygous site, of which 65% were loss-of-heterozygosity events. We excluded these sites from the comparative analysis because MuTect rejects sites where the normal appears to be contaminated or heterozygous, and SomaticSniper, Platypus and UnifiedGenotyper do not call SNVs on germline heterozygous sites. Overall, less than 0.9% of somatic events failed to reach convergence when multiSNV was allowed to run up to a maximum of 3000 Gibbs cycles. This was generally due to extremely low variant allele frequencies, and/or low depth. A summary of variant analysis results from multiSNV and the other variant callers is shown in Table 2.

**Table 2.** Summary of somatic variant calling statistics on the two datasets from (3) using SomaticSniper, MuTect, UnifiedGenotyper, Platypus and multiSNV and a combined approach using multiSNV and Platypus together

Method	Run time (min)	Somatic sites	Somatic SNVs	%age in dbSNP	Validated sensitivity (%)	Lowest VAF (%)
SomaticSniper	611	10 922	17 325	8.82	77.8	10.1
SomaticSniper (HC)	-	622	1 776	10.3	66.1	12.0
MuTect	13 100	274 792	871 741	32.6	96.5	2.78
MuTect (HC)	-	1 410	3 129	16.2	88.3	4.35
UnifiedGenotyper	2 428	6 294	23 638	10.1	90.1	7.69
Platypus	1 169	2 530	10 197	18.5	91.2	5.88
Platypus (HC)	-	796	3 968	25.0	90.1	5.88
multiSNV	2 174	21 115	85 809	6.93	97.1	2.78
multiSNV (HC)	429	7 712	20 872	6.15	97.1	2.78
multiSNV (HC) + Platypus	-	840	3 796	15.0	97.1	2.78

Filters were used whenever they were available for the given variant caller to identify a high confidence cohort of calls (denoted by HC). dbSNP concordance was computed using NCBI dbSNP Build 137, excluding sites after 129. The last three columns show how well each variant caller did on the validated SNVs. Sensitivity was calculated by dividing the total number of true positives by the total number of SNVs that were validated. The last column shows the SNV with the lowest variant allele frequency that was detected by each variant caller.

Platypus called the fewest somatic sites (2530) when no filtering was applied, followed by UnifiedGenotyper (6294). To get a high confidence dataset from Platypus, we kept only sites that were flagged as *‘alleleBiased’* and *‘PASS’*, which reduced the number of sites by more than three times. Similarly, when we used the publicly available scripts suggested for filtering SomaticSniper (Supplementary Material), the raw number of somatic sites dropped by more than 17 times, down to 622.

We applied simple filters to the output of multiSNV, as described in the Supplementary Material. This reduced the number of somatic sites called from 21 504 to 7736. MuTect found the most somatic sites (274 792); however, only 1410 satisfied the six built-in filters and were included in its high confidence (HC) calls.

#### Overlap with dbSNP

A frequent source of false positives in somatic variant analysis is germline variation being miscalled as somatic. We compared the overlap of somatic calls made by each variant caller with dbSNP build 137 (excluding sites after build 129). It is likely that not all sites called as somatic and found in dbSNP are false positives; however, on average this is a good measure of the false-positive rate. multiSNV had the lowest overlap with dbSNP on both unfiltered (6.93%) and high confidence (6.15%) mode, followed by SomaticSniper (8.82%).

#### Sensitivity in calling validated somatic sites and SNVs

We used the set of validated mutations to compare how many of the validated somatic sites and SNVs were called by each method. Overall, 27 SNV sites from patient 1 and 17 SNV sites from patient 2 were originally validated. multiSNV (in both modes) and UnifiedGenotyper called all SNV sites, whereas SomaticSniper missed three, even when no filters were applied. MuTect and Platypus in the unfiltered mode called all sites, but in both cases two were then rejected by the built-in filters.

SNVs were validated in several patient samples at these somatic sites, as reported in the Supplementary Material of (3). Out of 432 candidate SNVs, 171 were found to be genuine somatic variants. We compared the ‘validated sensitivity’ of the five methods by computing the number of validated SNVs called divided by the total number of validated SNVs.

**Figure 2.**
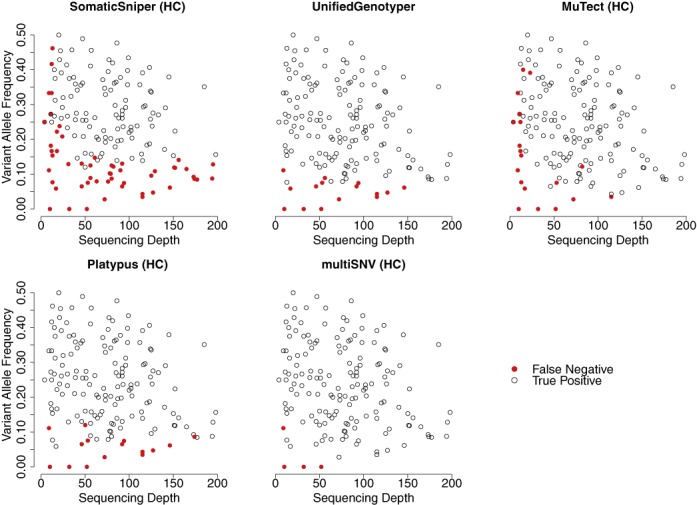
Validated SNVs from exome-sequencing data of patient 1 and patient 2 plotted as a function of the depth of coverage and variant allele frequency. SomaticSniper has a large number of false negatives even at relatively very high depths of coverage (>150). multiSNV has only five false negatives and of these four have 0 variant allele frequency in the sequencing reads. Some points on the plots overlie each other.

Figure 2 shows all true-positive and false-negative SNVs plotted as a function of sequencing depth and variant allele frequency, from reads filtered at mapping quality Q30 and base quality Q20. Out of 171 validated SNVs, multiSNV had five false negatives of which four were present at observed allele frequencies of 0, indicating that these sites could only be identified by subsequent validation experiments. Compared to MuTect (HC) and SomaticSniper (HC), UnifiedGenotyper, Platypus and multiSNV can more readily call SNVs at low sequencing depth as they consider multiple samples jointly, which improves power. The majority of false negatives with UnifiedGenotyper and Platypus were the result of very low variant allele frequencies, which appeared even at relatively high sequencing depth. This is because these tools are not somatic variant callers as such, so they lack the sensitivity to rare variants of tools like MuTect. SomaticSniper had the most false negatives and these appeared both at low sequencing depth and low variant allele frequency. In general, the performance of SomaticSniper is compromised compared to MuTect because it assumes that variant allele frequencies are close to 0.5.

As seen from Figure 2, multiSNV outperformed all other tools in terms of having the least number of false negatives, and was the only method whose validated sensitivity did not decrease after applying filters. By leveraging information from multiple samples, multiSNV was able to detect somatic mutations appearing at allele frequencies of 2.8% compared to 4.4% for MuTect, 5.9% for Platypus, 7.7% for UnifiedGenotyper and 12.0% for SomaticSniper.

#### A consensus approach

Platypus is unique in the cohort of variant callers considered, as it uses local *de novo* assembly, local realignment and probabilistic haplotype estimation, and it jointly calls indels, complex polymorphisms and SNPs (15). Unlike other variant callers, it has been shown to achieve high specificity without the need for extensive false-positive filtering. Compared to multiSNV, Platypus achieved a lower validated sensitivity (90.1 versus 97.1%), while calling 10 × fewer somatic sites. Thus, even though multiSNV is superior in correctly detecting the regional distribution of mutations at somatic sites, Platypus is more robust to artefactual variation, so a combined approach can leverage on the strengths of the two algorithms.

To get a high confidence dataset from multiSNV and Platypus, we retained somatic sites (genomic loci not individual calls) that were called by both multiSNV and Platypus, but used multiSNV to call the somatic status over each sample. The consensus approach helps remove false-positive sites appearing due to misalignments and other correlated errors, while the more sensitive multiSNV method identifies the regional distribution of mutations. This approach generates the top performing caller––matching the sensitivity of multiSNV while reducing the original set of somatic sites by almost 10 times.

## DISCUSSION

Several prominent cancer studies have started sequencing multiple tumour samples from the same patient to dissect the intratumour heterogeneity of cancer. This creates a need for a somatic variant caller that extends the Bayesian approaches used in the analysis of matched tumour-normal pairs to joint analysis of multiple same-patient samples. The main challenge is to model the joint distribution of genotypes (allelic compositions) with no information about the phylogenetic relationship between the tumour samples. This is trivial for tumour-normal pairs, as it is reasonable to assume that the (heterogeneous) tumour sample has evolved from a homogeneous normal sample. In the case of multiple, heterogeneous tumour samples, it is not trivial to infer the conditional dependencies between the samples. Even in the case where we have primary and metastatic samples, we may not assume that a mutation present in the primary sample also exists in the metastatic one, as the given mutation might have come from a clone present in the primary sample, and not ancestral to any of the clones in the metastasis.

In this first version of a multisample somatic caller, we assume samples are independent. More elaborate models could benefit from an ‘active learning’ approach, so that the regional distribution of calls is used to learn dependency structures and update the priors––this can in turn enhance the variant calling. Although powerful, such a model runs the risk of letting false-positive calls update the priors––this might falsely bias the variant calling and the inferred dependencies. Independent modelling simplifies statistical modelling and it also allows us to view any inferred dependence structure as unbiased, as sample dependencies are not used to call SNVs. It also allows us to make confidence statements about calls, for example when two samples are found to be empirically similar to one another, we can be more confident in calls made in one sample, if they are also seen in the second sample.

Aside being easily generalized to an arbitrary number of samples, multiSNV also addresses several of the issues that complicate variant calling from sequencing data. It is more flexible than some of the current pairwise somatic variant callers as it is able to identify SNVs at locations that are germline heterozygous or where no allele matches the reference. In addition, there is a built-in strand-bias test that pools reads from all somatic samples to increase the number of reads before applying Fisher's exact test. This is a more powerful approach than testing for strand-bias independently and it also has the advantage of generalizing to multiallelic sites. multiSNV is particularly good at detecting SNVs at low variant allele frequencies. This is because multiSNV calls SNVs by identifying the most likely allelic composition of each sample based on a likelihood model that is flexible enough to capture the effects of somatic variation by allowing for nondiploid genomes and an arbitrarily low variant allele frequency. As a result, sensitivity to call somatic variants depends more on the depth of coverage and less on the variant allele frequency. Furthermore, whenever an SNV is shared between multiple samples, multiSNV will use this information to update the prior probability of observing an SNV in a particular sample, based on how frequent that SNV is in the other same-patient samples. As a result, multiSNV has an enhanced ability to resolve low variant-allele frequencies, particularly in cases where the SNV is present at higher allele frequencies in other samples. A downside of this behaviour is that multiSNV will be susceptible to correlated sequencing artefacts such as context-related sequencing errors (for example at homopolymers) or misalignment due to the presence of indels, where ‘false-positive’ variant reads will be observed across different samples.

We compared multiSNV against SomaticSniper and MuTect, two widely used pairwise somatic variant callers, as well as UnifiedGenotyper and Platypus, which are currently the only available multisample variant callers. We used both simulated and real datasets. In simulations, most variant callers showed reasonable robustness to independent sequencing noise and multiSNV was one of the better algorithms in this aspect. Moreover, when we assessed algorithms based on their sensitivity to detect mutations present in multiple tumour samples, we found that the joint analysis approach in multiSNV consistently outperformed all other variant callers, particularly the pairwise callers (SomaticSniper and MuTect), especially when mutations were present at lower allele frequencies.

We used the multiregion exome sequencing datasets from (3) to test multiSNV and the four other variant callers using real data. Where available, we applied tool-specific filters to reduce the number of calls to a high confidence set. We found that somatic sites called by multiSNV had the lowest concordance with dbSNP, both with and without applying filters. MuTect and Platypus had the highest overlap with dbSNP, suggesting they might be more prone to miscalling germline variation as somatic.

multiSNV had the highest sensitivity in correctly identifying the subset of validated SNVs from (3), detecting 97% of all reported SNVs, in both filtered and unfiltered mode. It reported only five false negatives, of which four had observed variant allele frequency of 0 in the sequencing data. The UnifiedGenotyper identified 90% of all validated SNVs while calling more SNVs than the high confidence dataset from multiSNV. After filtering MuTect's output with the built-in filters, the number of calls became comparable to that of SomaticSniper after filtering, but the sensitivity of MuTect was 27.9% higher. This is an expected result as MuTect is a more recent variant caller that has addressed issues faced by earlier somatic variant callers. Platypus achieved a relatively good sensitivity (90.1%) and could detect mutations down to variant allele frequencies of 5.9%, while being one of the most conservative methods in its overall number of calls. As the emphasis of multiSNV is calling the correct regional distribution of mutations rather than detecting misalignments and other artefactual variation, we show that an optimal approach would be to filter somatic sites called by multiSNV using Platypus, which specifically addresses these issues. In future work, we aim to further develop the built-in false-positive filters, but even so, it is likely that such integrative approaches will outperform any single method.

## CONCLUSION

Our simulation and whole-exome sequencing analysis results demonstrate that multisample somatic variant calling can outperform independent or pairwise independent analyses of multiple related samples. In particular, we show that multiSNV will identify the correct regional distribution of mutations down to variant allele frequencies of 2.78%, while false-positive filtering and consensus approaches can reduce the overall number of calls with no adverse effect on the known number of false negatives. We anticipate that multisample somatic variant calling will become the gold standard when analysing NGS data from multiregion sequencing studies with spatially separated samples and longitudinal studies with samples collected at different time-points to track cancer evolution.

## SUPPLEMENTARY DATA

Supplementary Data are available at NAR Online.

SUPPLEMENTARY DATA
