# Supplementary material for: multiSNV: a probabilistic approach for improving detection of somatic point mutations from multiple related tumour samples
Source: Nucleic Acids Res. 2015 Feb 26;43(9):e61. doi: 10.1093/nar/gkv135 (PMC4482059; doi:10.1093/nar/gkv135)
Supplement: SUPPLEMENTARY DATA [file supp_43_9_e61__index.html]

multiSNV: a probabilistic approach for improving detection of somatic point mutations from multiple related tumour samples — SUPPLEMENTARY DATA 

# multiSNV: a probabilistic approach for improving detection of somatic point mutations from multiple related tumour samples

## SUPPLEMENTARY DATA

**Files in this Data Supplement:**

- Supplementary Data
