# Supplementary material for: multiSNV: a probabilistic approach for improving detection of somatic point mutations from multiple related tumour samples
Source: Nucleic Acids Res. 2015 Feb 26;43(9):e61. doi: 10.1093/nar/gkv135 (PMC4482059; doi:10.1093/nar/gkv135)
Supplement: SUPPLEMENTARY DATA [file supp_gkv135_nar-03043-met-k-2014-File008.pdf]

Supplementary Data

1 Software Package

multiSNV is open-source freely available software written in C++. It is currently compatible with SAMtools' multi-pileup format files and a BAM file compatible version will be released soon. Each line in the pileup file corresponds to a chromosomal location with columns that report chromosome and sequence number, reference allele, depth of coverage, read bases and read qualities for the normal sample followed by all tumour samples. multiSNV scans each multi-pileup line and runs the inference model on every location independently. Somatic events are reported in a standard VCF file that explicitly states the somatic status in each patient sample. The multiSNV workflow is shown in Figure 1.

1.1 Gibbs sampling

To infer the most likely set of allelic compositions $\{S^N, S^{T_1} \ldots S^{T_n}\}$, Gibbs sampling is used. This is a Markov Chain Monte Carlo method that allows us to approximate the posterior distribution $P(S^N, S^{T_1}, \ldots, S^{T_n}|\mathcal{D})$ by iteratively sampling from full conditionals that are easier to derive, as shown in Algorithm 1. We infer as a point estimate of each S, the most frequently drawn state. We also report the 'quality' of each estimate, represented by the Phred-scaled ratio of the frequency of the two most commonly drawn values of S. The number of Gibbs iterations is set to 600, and a thinning parameter of 5 is applied to reduce the autocorrelation of the Markov chain.

1.1.1 Assessing convergence

Markov Chain Monte Carlo methods are known to suffer from convergence issues particularly in the presence of multiple local minima. Assessing convergence of the Gibbs sampler is optional in multiSNV and conducted by using Fisher's exact test to determine whether values drawn from earlier and later parts of the chain (excluding burn-in period) appear to come from the same (stationary) distribution. If the convergence criterion is not met, we

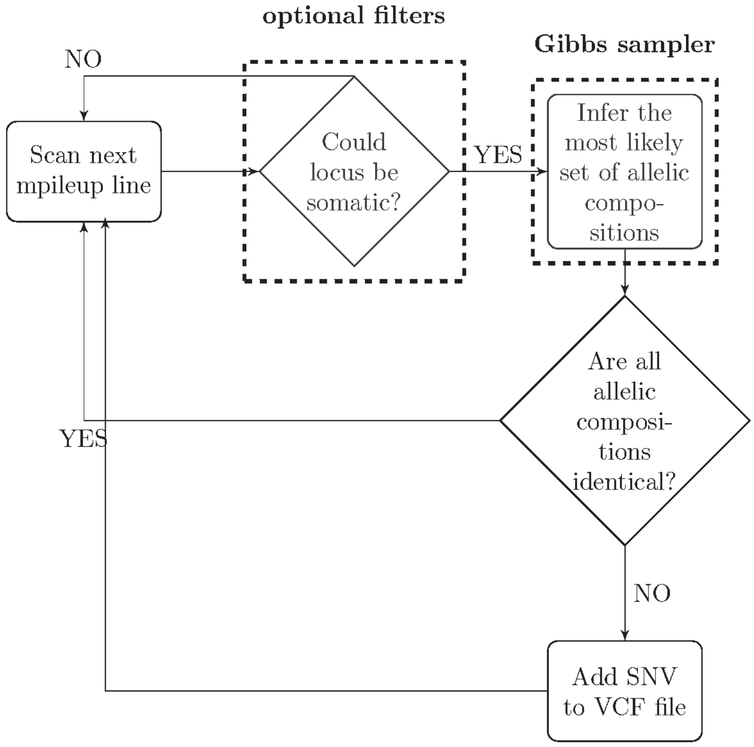

Figure 1: The flow diagram summarises the key routines of multiSNV. Each mpileup line holds information about the sequencing reads and qualities in each sample at a particular chromosomal locus. The 'optional filters' can help reduce overall run time by conducting inference only in cases of interest or where there is indication of somatic variation.

continue running the Gibbs sampler until the maximum allowed number of Gibbs cycles is reached (set to 3000). In the rare cases where the Gibbs sampler reaches the maximum number of iterations and still fails to converge, the corresponding VCF file entry is flagged accordingly (MAX_ITER). Fisher's exact test is computationally expensive for the number of states specified (14) hence this optional step can increase running time by up to a factor of three.

1.2 Strand-bias test

Strand bias occurs when the distribution of reference-variant reads on the forward and reverse strand is significantly different. Such cases are of interest as strand bias is a common feature of false positive somatic calls. multiSNV

Algorithm 1: Gibbs sampling in multiSNV

```
Initialize S_N^0, S_{T_1}^0 ... S_{T_n}^0;
converge ← false;
i ← 0;
while converge == false and i ≤ MAX do
    for k ← 1 to T do
        sample S_N^k from P(S_N | S_{T_1}^{k-1}, ..., S_{T_n}^{k-1}, D);
        sample S_{T_1}^k from P(S_{T_1} | S_{T_1}^{k-1}, ..., S_{T_n}^{k-1}, S_N^k, D) ;
        ⋮
        sample S_{T_n}^k from P(S_{T_n} | S_{T_{-n}}^k, S_N^k, D) ;
        i ← i + 1;
    end
    converge ← IsConverged(S_N^{b:i-1}, S_{T_1}^{b:i-1}, ..., S_{T_n}^{b:i-1}) ;  // b is the
    burn-in period, samples 0 to b are discarded
end
```

uses Fisher's exact test to identify sites with significant strand-bias similarly to [1] but can also generalise to multi-allelic sites. In addition, the built-in strand bias test takes advantage of the multiple samples and pools reads from all samples found to be somatic, so that it improves power to identify sites with significant strand bias. These sites are flagged accordingly in the VCF file.

1.3 Optional filters to reduce run time

Several factors affect run time, including the number of patient samples to be analysed, the number of pileup lines, the number of Gibbs iterations and whether convergence checks need to be performed. Since the inference step is the most computationally expensive part of multiSNV, we note that we can greatly improve run time by using a set of user-specified criteria to 'select' the chromosomal locations to run inference on. Sites of interest include locations with some minor indication of variation, i.e. sites with some minimum number of mismatch reads found. We can also optionally select to ignore sites that fail to meet minimum and maximum coverage criteria. These filters are summarised in Fig. 2.

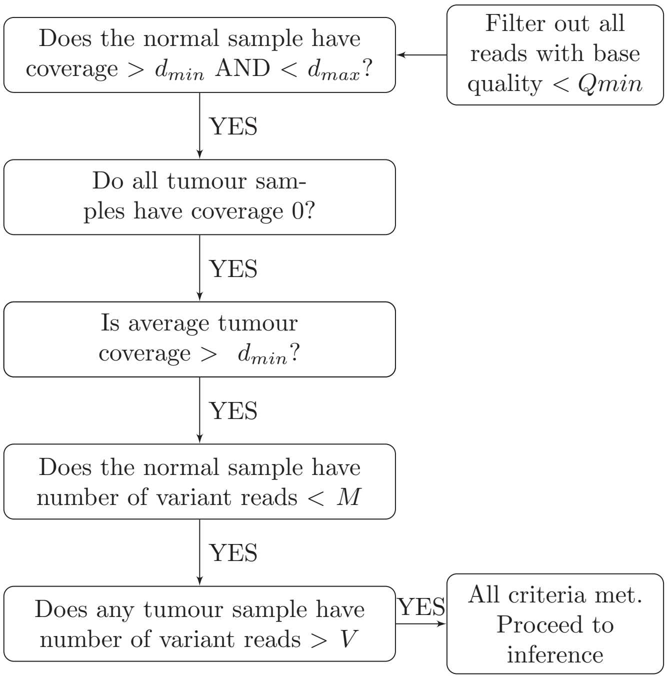

Figure 2: Optional filters used to reduce run time by performing inference on sites that meet certain user-specified criteria.

2 Using multiSNV

Further information about compiling and using multiSNV may be found at `http://www.compbio.group.cam.ac.uk/software/multisnv`

3 Simulations

3.1 Generating BAM files for simulations

We used the SimulateReadsForVariants tool from GATK to generate BAM files with the readSamplingMode option set to 'constant' and setting the nonDeterministicRandomSeed option to true. The readDepth and errorRate options were varied as required by each experiment and the remaining settings were set to their default values. We simulated BAM files from four tumour samples and one normal sample.

A sample VCF file with $1,000$ sites was used as input. This generated BAM files with reads covering about $100,000$ loci (simulated read length was left at the default value of 101). Since the tool generates sites with variant allele frequency of 0.5 or 0 depending on the genotypes in the VCF file, we merged BAM files and then downsampled to the target depth to simulate lower variant allele frequencies and to add tumour contamination to the normal sample.

3.2 Settings used

We tried to set settings as close as possible to their default values. For all algorithms we set the minimum base quality to 10 as we wanted to disable filtering of simulated reads. For Platypus we additionally disabled filtering of duplicates (most simulated reads are duplicates) and merging of clustered variants (to make the number of calls comparable). For multiSNV we also set the median depth in normal and tumour to the simulated read depth.

4 Clear-cell renal carcinoma data analysis

4.1 Settings used

We aligned fastq files with bwa 0.7.4 using the same settings as the original analysis.

4.1.1 multiSNV

The pileup files required by multiSNV were produced with the `mpileup` command from Samtools using BAQ computation, a minimum mapping quality of 30 and leaving other settings at default values. We ran multiSNV twice, once using values close to multiSNV's default rules for determining which genomic locations should be considered, and once using a 'brute-force' approach where effectively each genomic location was considered. The values used in each case are shown on Table 1. Since we were not concerned with reducing run time we also ran convergence checks on the Gibbs sampler. The high confidence dataset was then filtered to exclude sites flagged for strand bias or convergence failure and sites clustering within 25bp of each other. All germline heterozygous sites were filtered out.

4.1.2 SomaticSniper

We ran SomaticSniper in the joint mode specifying a minimum mapping quality of 30 for reads and leaving other settings as default. To obtain the high confidence dataset (SomaticSniper HC) we used the publicly available Perl scripts (fpfilter.pl and highconfidence.pl) specifying the minimum acceptable base quality (b) as 15, the minimum acceptable mapping quality (q) as 30 and leaving other settings at their default values. Output files were merged using the `CombineVariants` tool from GATK.

4.1.3 UnifiedGenotyper

UnifiedGenotyper was ran at default values, specifying a minimum base quality of 20. Events on germline heterozygous sites were filtered out.

4.1.4 MuTect

We ran MuTect at default values. The high confidence dataset (MuTect HC) consisted of all sites that were not flagged as REJECT in the output VCF file.

4.1.5 Platypus

We ran Platypus using minimum mapping base quality of 30, minimum base quality of 20, strand bias threshold of 0.01, filtering duplicates *–minReads 1* and leaving other settings at default. As with multiSNV, all germline heterozygous sites were filtered out. To get a high confidence dataset from Platypus we kept only sites that were flagged as *'alleleBiased'* and *'PASS'*.

4.2 Comments

In all cases only sites with non-zero depth in all samples were considered. All loss of heterozygosity (LOH), germline heterozygous sites and multi-allelic sites were excluded from the output to make results comparable. Variant calling was conducted on the same 8-core 2.4GHz machine. To analyse the two datasets using SomaticSniper and MuTect we had to run each tool 17 times, as all 17 tumour exomes had to be analysed independently with their corresponding normal sample. multiSNV and the UnifiedGenotyper were run once on each dataset using all exomes from the same patient.

Option	Description	Default Value	CCRC	CCRC HC
V	As shown on Fig. 2	2	**1**	2
M	As shown on Fig. 2	100	**10000**	**1**
d_{min}	As shown on Fig. 2	5	**1**	5
d_{max}	As shown on Fig. 2	300	**25000**	300
$medianT$	Median coverage in tumour samples	40	40	40
$medianN$	Median coverage in normal sample	40	40	40
m	Mutation rate	3×10^{-7}	3×10^{-7}	3×10^{-7}
Q	Minimum acceptable base quality	20	20	20
$conv$	Check for convergence	false	**true**	**true**

Table 1: List of multiSNV command-line options with default values. The fourth and fifth column indicate values used to analyse the CCRC dataset in brute-force and high confidence mode, respectively.

References

[1] Larson, D.E., Harris, C.C., Chen, K., Koboldt, D.C., Abbott, T.E., Dooling, D.J., Ley, T.J., Mardis, E.R., Wilson, R.K. and Ding, L. (2012) SomaticSniper: identification of somatic point mutations in whole genome sequencing data. *Bioinformatics*, **28**, 311–317.
